# Prognostic value of NT-proBNP in the new era of heart failure treatment

**DOI:** 10.1371/journal.pone.0309948

**Published:** 2024-09-13

**Authors:** Dat Vu Nguyen, Si Van Nguyen, An Le Pham, Bay Thi Nguyen, Sy Van Hoang

**Affiliations:** 1 University of Medicine and Pharmacy at Ho Chi Minh City, Ho Chi Minh City, Vietnam; 2 Nguyen Tri Phuong Hospital, Ho Chi Minh City, Vietnam; 3 School of Medicine, Vietnam National University Ho Chi Minh City, Ho Chi Minh City, Vietnam; 4 Cho Ray Hospital, Ho Chi Minh City, Vietnam; PearResearch / Government Doon Medical College, INDIA

## Abstract

**Background:**

Heart failure is one of the leading causes of mortality and hospitalization in cardiovascular patients. Guideline-directed medical treatment (GDMT) in the current era includes novel medications such as ARNI and SGLT2 inhibitors, as well as an approach to treatment based on clinical phenotypes. To assess prognostic factors for mortality and hospital readmissions plays a crucial role in patient care.

**Objectives:**

This study aimed to determine the rate of 90-day post-discharge events in patients having heart failure with reduced ejection fraction (HFrEF) and investigate the associated clinical factors.

**Method:**

A prospective study was conducted on 110 HFrEF patients at the cardiology department of Cho Ray Hospital. The 90-day events included all-cause mortality and rehospitalization due to heart failure.

**Results:**

The rate of 90-day events was 45.6%. After multivariable Cox regression analysis, NT-proBNP level ≥ 1858 pg/mL was identified as an independent factor associated with the 90-day events.

**Conclusion:**

NT-proBNP cut-off ≥ 1858 pg/mL can be used for the prognosis of 90-day events in HFrEF.

## Introduction

Heart failure is one of the leading causes of mortality and hospitalization among cardiac patients. A real-world study showed a 22.5% mortality rate within two years in heart failure patients with reduced left ventricular ejection fraction, and approximately 56.0% of patients were readmitted due to heart failure within 30 days of heart failure events [1]. Therefore, accurately identifying prognostic factors for readmission and mortality in heart failure patients can help clinicians detect high-risk patients early. This becomes even more significant as current heart failure treatment is evolving with novel medications such as ARNI and SGLT2 inhibitors, as well as a shift towards a clinical phenotype-based rather than stepwise approach [2].

## Method

### Patients

This prospective study enrolled 110 patients with heart failure with reduced ejection fraction (HFrEF) who were admitted to Cho Ray Hospital where the recruitment time was from December 1, 2022 to June 30, 2023. Patients who refused to join the study were excluded from the analysis. Since atrial fibrillation can influence NT-proBNP levels significantly, those with this condition were not included. Patients’ clinical characteristics are presented in Table 1.

**Table 1:** Baseline characteristics.

	All patients	Non-event	Event	P-value
	(N = 110)	(N = 60)	(N = 50)	
**Age (years)**	61.9 ± 16.0	61.9 ± 16.6	61.8 ± 15.3	0.959
**Female**	42 (38.2)	26 (43.3)	16 (32.0)	0.220
**BMI (kg/m** ^**2**^ **)**	22.4 ± 2.6	22.4 ± 2.3	22.4 ± 2.9	0.847
**Hypertension**	96 (87.3)	55 (91.6)	41 (82.0)	0.130
**Type 2 diabetes**	45 (40.9)	24 (40.0)	21 (42.0)	0.848
**Dyslipidemia**	95 (86.3)	53 (88.3)	42 (84.0)	0.583
**Chronic lung disease**	3 (2.7)	1 (1.7)	2 (4.0)	0.590
**Stroke**	6 (5.5)	4 (6.7)	2 (4.0)	0.687
**Cause of heart failure**				
** Ischemia**	95 (86.4)	54 (90.0)	41 (82.0)	0.223
** Non-ischemia**	15 (13.6)	6 (10.0)	9 (18.0)	
**EF (%)**	27.5 ± 7.8	28 ± 7.7	26.9 ± 8.0	0.463
**Hemoglobin (g/dL)**	12.1 ± 2.2	12.2 ± 2.1	11.9 ± 2.3	0.395
**BUN (mg/dL)**	25.7 ± 15.4	21.9 ± 9.7	30.1 ± 19.3	**0.006**
**eGFR (mL/min/1.73m** ^**2**^ **)**	63.0 ± 27.5	67.9 ± 23.7	57.0 ± 30.6	**0.038**
**Serum sodium (mEq/L)**	136.3 ± 4.9	136.8 ± 4.8	135.7 ± 4.9	0.251
**NT-proBNP (pg/mL)**	1395	1962	2400	**0.004**
	(841–2620)	(763–1845)	(916–5398)	
**Medications**				
** ARNI/ACEIs/ARBs**	90 (81.8)	54 (90.0)	36 (72.0)	**0.015**
** Beta blockers**	58 (52.7)	35 (58.3)	23 (46.0)	0.197
** MRA**	87 (79.1)	51 (85.0)	36 (72.0)	0.095
** SGLT2 inhibitors**	82 (74.5)	52 (86.7)	30 (60.0)	**0.001**
** Loop diuretic**	53 (48.2)	28 (46.7)	25 (50.0)	0.728

BMI: Body mass index, EF: Ejection fraction, BUN: Blood urea nitrogen, eGFR: estimated Glomerular filtration rate (CKD-EPI), NT-proBNP: N-terminal pro B-type natriuretic peptide, ARNI: Sacubitril/Valsartan, ACEI: Angiotensin converting enzyme inhibitor, ARB: Angiotensin receptor blocker, MRA: Mineralocorticoid receptor antagonist, SGLT2: Sodium glucose cotransporter 2.

HFrEF was defined as patients exhibiting clinical symptoms consistent with heart failure and left ventricular ejection fraction (EF) ≤ 40% measured by echocardiography using Simpson’s biplane method [2]. EF was performed by cardiologists certified in echocardiography at the cardiology department of Cho Ray Hospital using Vivid E95 ultrasound machines, GE Healthcare. The probe was placed at the apex of the heart pointing towards the base of the heart, selecting a four-chamber view from the apex at end-diastolic and end-systolic phases. The border of the left ventricular endocardium was traced. The machine automatically calculated end-diastolic and end-systolic volume and EF. The measured values as described above in the two-chamber view were deployed to calculate the biplane EF [3].

NT-proBNP was measured by the commercial sandwich electrochemiluminescence Elecsys proBNP II immunoassay using a Cobas e 602 analyzer (Roche Diagnostics) according to the manufacturer’s instructions. NT-proBNP, serum sodium concentration, hemoglobin, BUN (blood urea nitrogen), and serum creatinine were measured at the biochemical department of Cho Ray Hospital and taken one day before discharge. Quality control for laboratory testing complies with the regulations of the Vietnam Ministry of Health.

The study protocol received approval from the Ethics Committee of the University of Medicine and Pharmacy at Ho Chi Minh City (No. 830/IRD/UMP, November 3, 2022). Written informed consent was obtained from all patients before their enrollment. The investigation complied with the principles outlined in the 1975 Declaration of Helsinki.

### Follow-up

Post-discharge outcomes include all-cause mortality and heart failure readmission confirmed by direct interviews or phone calls. The duration of the follow-up period ranged from 16 to 238 days (median 99 days).

### Data analysis

All descriptive data are presented as mean ± SD (standard deviation), median, or frequency (%). The Shapiro-Wilk test revealed that NT-proBNP was not normally distributed, so it is shown as the median and interquartile range (25th and 75th percentiles). Continuous variables between the two groups were compared using either the unpaired t-test or the Mann-Whitney U-test, as appropriate. Frequencies were compared using the Chi-squared test. Receiver operating characteristic (ROC) curve analysis was employed to determine the NT-proBNP cut-off. The predictive potential was evaluated using univariate and multivariate Cox proportional hazards analyses. Multivariate Cox proportional hazards analysis included confounders that were significant in the univariate model. Both univariate and multivariate Cox proportional hazards analyses investigated 1-SD changes in continuous variables. Kaplan-Meier survival analysis was utilized to compare the two groups using the NT-proBNP cut-off.

All probabilities were expressed as two-tailed, with statistical significance defined as P < 0.05. All confidence intervals were calculated at the 95% level. Statistical analysis was performed using STATA 20.0 (StataCorp, College Station, TX, USA).

## Results

A total of 110 HFrEF patients completed the follow-up study without any dropped-out cases. During the follow-up period, 50 patients had a cardiovascular event that included 8 all-cause deaths and 42 readmissions due to heart failure.

### Baseline characteristics

Compared with patients without 90-day events, those with events had lower eGFR and increased NT-proBNP and BUN at baseline. There was no significant difference in EF between the 2 groups. ARNI/ACEIs/ARBs and SGLT2 inhibitors were prescribed at lower rates in the event group (Table 1).

### Predictive value of NT-proBNP

**Fig 1:**
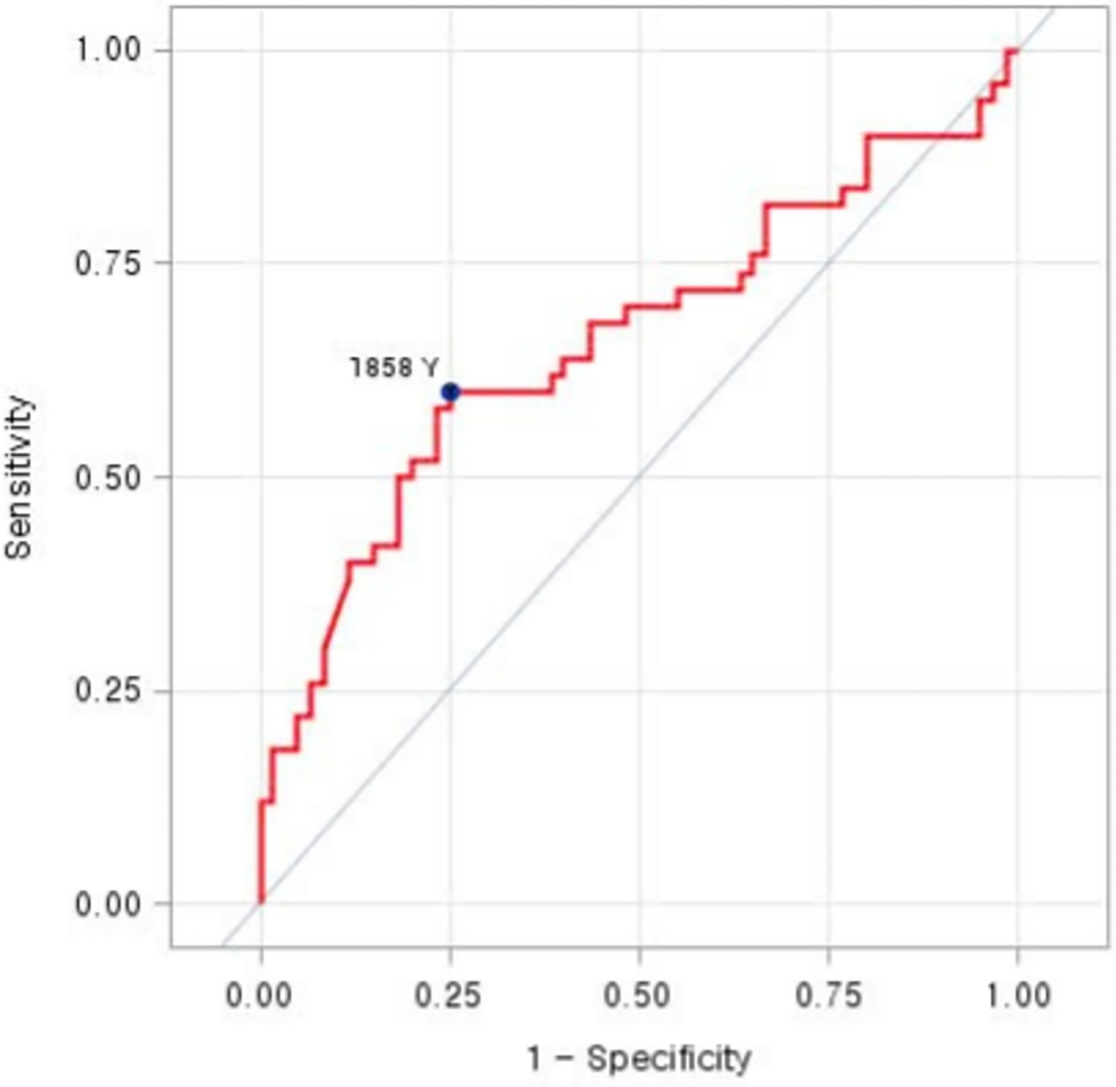
Area under the ROC curve of NT-proBNP in predicting 90-day events. AUC = 0.66, sensitivity = 60.0%, specificity = 75.0% and accuracy = 68.0%.

The NT-proBNP cut-off of 1858 pg/mL, determined through ROC curve analysis, provided an AUC of 0.66, sensitivity of 60.0%, specificity of 75.0%, and accuracy of 68.0% for predicting 90-day events (Fig 1).

**Fig 2:**
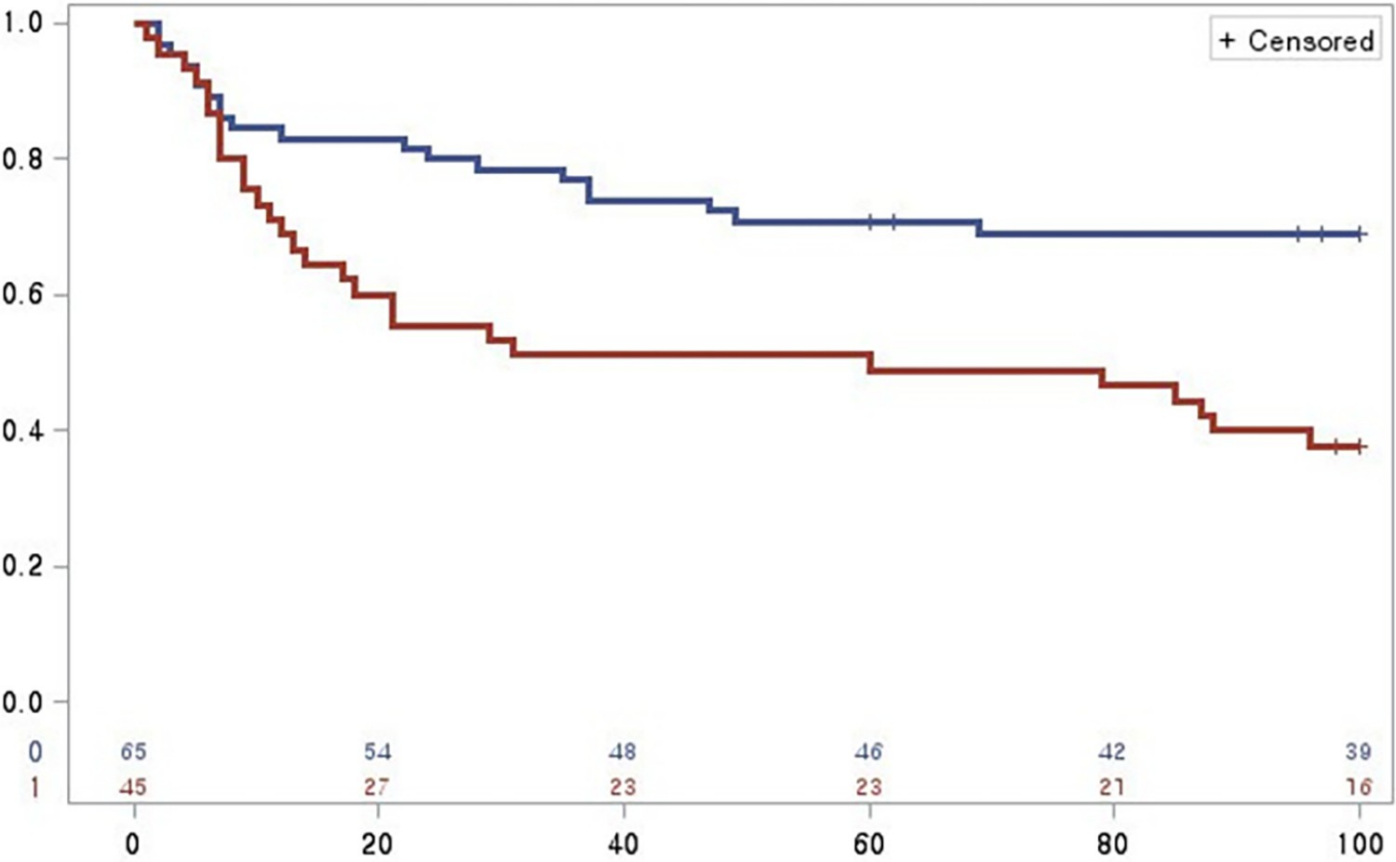
Kaplan-Meier curve of 90-day events according to NT-proBNP strata (the cut-off point is 1858 pg/mL. The X-axis represents time to event (days) and the Y-axis represents event-free probability. The red line (NT-proBNP ≥ 1858 pg/mL) indicates lower event-free probability after 90 days compared to the blue line (NT-proBNP < 1858 pg/mL), P log-rank = 0.0005.

When patients were stratified into two groups based on the NT-proBNP cut-off, Kaplan-Meier analysis showed that those with high NT-proBNP levels (≥ 1858 pg/mL) had a higher probability of future cardiovascular events compared to those with lower levels (< 1858 pg/mL; P = 0.0005, log-rank test). Notably, the divergence of the two curves occurred very early. Indeed, after a few days, there was a very large difference in the event rates between two groups (Fig 2). As indicated in Table 2, patients with higher NT-proBNP levels experienced more frequent all-cause deaths and rehospitalizations than those with lower NT-proBNP levels.

**Table 2:** Baseline characteristics vs. NT-proBNP levels.

	NT-proBNP < 1858 pg/mL	NT-proBNP ≥ 1858 pg/mL	P-value
	(N = 65)	(N = 45)	
**Age (years)**	60.3 ± 16.1	64.0 ± 15.7	0.236
**Female**	22 (33.8)	20 (36.4)	0.261
**BMI (kg/m** ^**2**^ **)**	22.1 ± 2.4	22.7 ± 2.8	0.553
**Cause of heart failure**			
** Ischemia**	57 (87.7)	41 (84.4)	0.626
** Non-ischemia**	8 (12.3)	9 (15.6)	
**Hypertension**	59 (90.8)	37 (82.2)	0.816
**Type 2 diabetes**	26 (40.0)	19 (42.2)	0.816
**Dyslipidemia**	56 (86.2)	39 (86.7)	0.939
**Chronic lung disease**	3 (4.6)	0 (0)	0.268
**Stroke**	5 (7.7)	1 (2.2)	0.398
**EF (%)**	28.3 ± 7.5	26.5 ± 8.2	0.236
**Hemoglobin (g/dL)**	12.7 ± 2.0	11.2 ± 2.2	**0.001**
**BUN (mg/dL)**	23.4 ± 10.1	28.9 ± 20.4	0.186
**eGFR (mL/min/1.73m** ^**2**^ **)**	67.9 ± 23.7	57.0 ± 30.6	0.052
**Serum sodium (mEq/L)**	136.4 ± 4.9	136.2 ± 4.9	0.824
**NT-proBNP (pg/mL)**	896	4130	**< 0.001**
	(565–1257)	(2756–8946)	
**Medications**			
** ARNI/ACEIs/ARBs**	53 (81.5)	37 (82.2)	**0.927**
** Beta blockers**	37 (56.9)	21 (46.7)	0.257
** MRA**	56 (86.2)	31 (68.9)	**0.029**
** SGLT2 inhibitors**	51 (78.5)	31 (68.9)	0.257
** Loop diuretic**	31 (47.7)	22 (48.9)	0.902
**90-day events**	20 (30.8)	30 (66.7)	**< 0.001**

BMI: Body mass index, EF: Ejection fraction, BUN: Blood urea nitrogen, eGFR: estimated Glomerular filtration rate (CKD-EPI), NT-proBNP: N-terminal pro B-type natriuretic peptide, ARNI: Sacubitril/Valsartan, ACEI: Angiotensin converting enzyme inhibitor, ARB: Angiotensin receptor blocker, MRA: Mineralocorticoid receptor antagonist, SGLT2: Sodium glucose cotransporter 2.

Univariate Cox proportional hazards analysis revealed that ARNI/ACEIs/ARBs (HR 0.42; 95% CI: 10.23–0.79), SGLT2 inhibitors (HR 0.40; 95% CI: 0.23–0.70), eGFR (HR 0.99; 95% CI: 0.98–0.99), and NT-proBNP (≥ 1858 pg/mL, HR 2.99; 95% CI: 1.46–6.11) were significant predictors of 90-day events (Table 3). In multivariate Cox proportional hazards analysis, only high NT-proBNP (HR 2.36; 95% CI: 1.31–4.24) remained a significant predictor of 90-day events (Table 3).

**Table 3:** Univariate and multivariate Cox proportional hazard analysis for 90-day events.

	Univariate		Multivariate	
	HR (95%CI)	P-value	HR (95%CI)	P-value
**Female**	0.64 (0.35–1.16)	0.141	--	--
**Age**	0.87 (0.98–1.02)	0.999	--	--
**BMI**	1.01 (0.90–1.14)	0.842	--	--
**ARNI/ACEIs/ARBs**	0.42 (0.23–0.79)	**0.007**	0.49 (0.23–1.03)	0.060
**Beta blockers**	0.68 (0.39–1.19)	0.181	--	--
**SGLT2 inhibitors**	0.40 (0.23–0.70)	**0.001**	0.53 (0.27–1,02)	0.056
**MRA**	0.62 (0.33–0.15)	0.129	--	--
**Hemoglobin**	0.97 (0.86–1.10)	0.628	--	--
**eGFR**	0.99 (0.98–0.99)	**0.030**	0.86 (0,99–1,01)	0.863
**Serum sodium**	0.96 (0.91–1.02)	0.191	--	--
**EF**	0.99 (0.95–1.02)	0.460	--	--
**NT-proBNP ≥ 1858 pg/mL**	2.49 (1.41–4.40)	**0.002**	2.36 (1,31–4,24)	**0.004**

BMI: Body mass index, ARNI: Sacubitril/valsartan, ACEi: Angiotensin converting enzyme inhibitor, ARB: Angiotensin receptor blocker, SGLT2: Sodium glucose cotransporter 2, MRA: Mineralocorticoid antagonist, eGFR: estimated Glomerular filtration rate (CKD-EPI), EF: Ejection fraction, NT-proBNP: N-terminal pro B-type natriuretic peptide.

## Discussion

Our study population had a relatively high average age, with males predominating and a high prevalence of comorbidities. These results are consistent with similar local and international studies [4, 5]. This demonstrates the consistency in heart failure populations and suggests the challenges in managing heart failure related to patient characteristics. Specifically, advanced age is associated with declining organ function, while multiple comorbidities can lead to a burden of medication and drug interactions [5].

The medical treatment of heart failure according to recommendations in our study yielded quite favorable results, with over two-thirds of patients receiving guideline-directed medical therapy (GDMT) in pre-discharge prescriptions, except for the beta blockers. The cardiology department of Cho Ray tertiary hospital had to handle severe heart failure cases from various referral centers as well as a high volume of patients, which might affect beta-blocker initiation during the pre-discharge period. Initiating beta-blockers during the in-hospital phase has been shown to improve the prognosis for patients with HFrEF, as evidenced by the STRONG-HF study (The Safety, Tolerability, and Efficacy of Rapid Optimization, Helped by NT-proBNP Testing, of Heart Failure Therapies) [6]. The recommended beta-blockers for HFrEF include carvedilol, metoprolol, bisoprolol, and nebivolol. Importantly, for unstable patients, the risk of worsening heart failure after starting beta-blockers may be reduced by concurrently administering SGLT2 inhibitors, which are also strongly recommended for HFrEF [7].

The current GDMT includes novel drugs such as ARNI and SGLT2 inhibitors. TRANSITION and PIONEER-HF trials have shown that initiating ARNI during the inpatient period improves natriuretic peptide levels, thereby potentially reducing readmission and mortality [8, 9]. Furthermore, SGLT2 inhibitors have shown many benefits in stable acute heart failure, as demonstrated in the EMPULSE clinical trial [10]. Therefore, early initiation of these drugs is recommended in updated guidelines [11].

BNP (B-type natriuretic peptide) with a half-life of about 22 minutes may reflect changes in pulmonary congestion every 2 hours while NT-proBNP with a half-life of 120 minutes may reflect average hemodynamic changes in the body every 12 hours. Studies have consistently shown that the median values of these biomarkers are elevated in patients with HFrEF [12]. Both BNP and NT-proBNP decrease promptly and significantly in refractory heart failure when effectively treated with intravenous diuretics and nitroprusside during the acute phase [13]. When using classic drugs that affect the neurohormonal axis, such as beta blockers and ACEIs/ARBs, these biomarkers decrease, indicating a response to treatment [14, 15]. Besides, BNP-guided treatment of chronic heart failure can help reduce total mortality and hospitalization [16]. In our study, the group with events also had higher NT-proBNP levels than the other group.

In recent medical treatment of heart failure, the emergence of ARNI and SGLT2 inhibitors brings many positive effects and benefits for patients. ARNI intervenes in the degradation process of natriuretic peptides, leading to differences in BNP dynamics compared to NT-proBNP. Specifically, BNP increases after initiation while NT-proBNP decreases, and both phenomena can occur in patients with clinical improvement. Therefore, NT-proBNP tends to be selected for treatment evaluation rather than BNP during ARNI prescription, although the subsequent increase of both biomarkers indicates worsening heart failure [17]. Conversely, the mechanism of action of SGLT2 inhibitors in heart failure is not fully understood. Although the drug has diuretic effects, thereby potentially affecting hemodynamics, it does not fully explain the substantial benefits in prognosis. Regarding NT-proBNP, SGLT2 inhibitors still show inconsistent results in reducing the concentration of this biomarker upon prescription [18, 19]. Therefore, there may be other advantages beyond the neurohormonal axis mechanism of SGLT2 inhibitors [20]. This also explains the unique role of SGLT2 inhibitors in improving outcomes for heart failure with preserved ejection fraction, which has more complex pathophysiological mechanisms [21]. All of these raise the question if NT-proBNP remains predictive in such treatment innovations and our study helps demonstrate the value of NT-proBNP, independent of other factors in 90-day event prognosis. The cut-off value of 1858 pg/mL was higher than those of previous studies which of note, had a low rate of SGLT2 inhibitor and ARNI prescription [22, 23].

Our investigation reaffirmed the prognostic role of NT-proBNP in the new era of heart failure treatment and identified an applicable cut-off point for clinical use. However, we acknowledge some limitations in this study: (1) conducted at a single center, hence lacking representativeness, (2) not assessing NT-proBNP dynamics and medication adjustments in the post-discharge period and (3) excluding atrial fibrillation which is often encountered in HFrEF. These existing points should be addressed by more detailed similar multicenter studies.
